# Herpesvirus Telomerase RNA (vTR) with a Mutated Template Sequence Abrogates Herpesvirus-Induced Lymphomagenesis

**DOI:** 10.1371/journal.ppat.1002333

**Published:** 2011-10-27

**Authors:** Benedikt B. Kaufer, Sina Arndt, Sascha Trapp, Nikolaus Osterrieder, Keith W. Jarosinski

**Affiliations:** 1 Department of Microbiology and Immunology, College of Veterinary Medicine, Cornell University, Ithaca, New York, United States of America; 2 Institut für Virologie, Freie Universität Berlin, Berlin, Germany; University of North Carolina, United States of America

## Abstract

Telomerase reverse transcriptase (TERT) and telomerase RNA (TR) represent the enzymatically active components of telomerase. In the complex, TR provides the template for the addition of telomeric repeats to telomeres, a protective structure at the end of linear chromosomes. Human TR with a mutation in the template region has been previously shown to inhibit proliferation of cancer cells *in vitro*. In this report, we examined the effects of a mutation in the template of a virus encoded TR (vTR) on herpesvirus-induced tumorigenesis *in vivo*. For this purpose, we used the oncogenic avian herpesvirus Marek's disease virus (MDV) as a natural virus-host model for lymphomagenesis. We generated recombinant MDV in which the vTR template sequence was mutated from AATCCCAATC to A**TATAT**A**TAT** (vAU5) by two-step Red-mediated mutagenesis. Recombinant viruses harboring the template mutation replicated with kinetics comparable to parental and revertant viruses *in vitro*. However, mutation of the vTR template sequence completely abrogated virus-induced tumor formation *in vivo*, although the virus was able to undergo low-level lytic replication. To confirm that the absence of tumors was dependent on the presence of mutant vTR in the telomerase complex, a second mutation was introduced in vAU5 that targeted the P6.1 stem loop, a conserved region essential for vTR-TERT interaction. Absence of vTR-AU5 from the telomerase complex restored virus-induced lymphoma formation. To test if the attenuated vAU5 could be used as an effective vaccine against MDV, we performed vaccination-challenge studies and determined that vaccination with vAU5 completely protected chickens from lethal challenge with highly virulent MDV. Taken together, our results demonstrate 1) that mutation of the vTR template sequence can completely abrogate virus-induced tumorigenesis, likely by the inhibition of cancer cell proliferation, and 2) that this strategy could be used to generate novel vaccine candidates against virus-induced lymphoma.

## Author Summary

Telomerase facilitates telomere maintenance and consists of two major components: the catalytic subunit telomerase reverse transcriptase (TERT) and telomerase RNA (TR) that provides the template for the addition of telomeric repeats to telomere ends. Expression of TRs with a mutation in the template sequence can result in telomere instability, cell cycle arrest and apoptosis in mammalian cells. Here, we introduced a template mutation in a TR encoded by the highly oncogenic avian herpesvirus Marek's disease virus (MDV) to evaluate this mechanism in a natural virus-host model for virus-induced tumorigenesis. Expression of the mutant viral telomerase RNA (vTR) by MDV allowed virus replication in telomerase-deficient cells, but completely abrogated MDV-induced lymphomagenesis *in vivo* in a telomerase-dependent manner. Infection with MDV harboring the template mutation in vTR not only abrogated herpesvirus-induced tumorigenesis, but also protected chickens from lethal challenge with a very virulent MDV strain. We provide the first *in vivo* evidence that a TR with a template mutation expressed by a herpesvirus can be used to prevent herpesvirus-induced tumorigenesis, an approach that could be used for the development of the next generation vaccines against MDV and possibly also other lymphotropic herpesviruses.

## Introduction

**Figure 1:**
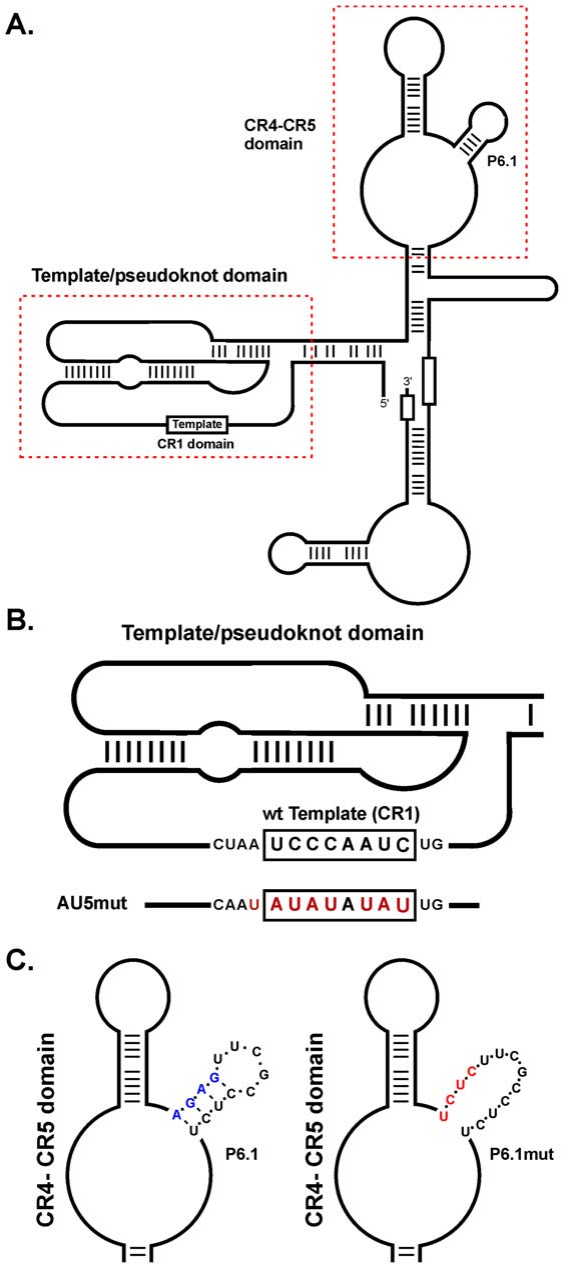
diagram of MDV vTR secondary structure, location of the CR1 and CR4-5 domains, and incorporated mutations. A) The pseudoknot (core), containing the template sequence, and the CR4-CR5 domains containing the P6.1 stem loop, are indicated with boxes. B) The pseudoknot domain including the sequence of wild-type vTR template and AU5 template mutant (AU5). Nucleotide changes in the template sequence are shown in red. C) The CR4-CR5 domain showing detailed representations of the P6.1 stem-loop and the structures of wild-type P6.1 (left) and mutant P6.1 stem-loop (P6.1mut) (right) are shown. Nucleotide changes of the wt P6.1 stem-loop (blue) are shown in red and have been previously published [22].

Telomerase is a multi-component ribonucleoprotein complex that governs the maintenance of telomeres, protein-associated hexameric sequence repeats at the end of linear chromosomes, and ensures chromosomal integrity and cellular survival [1], [2]. The telomerase complex consists of two core components, telomerase reverse transcriptase (TERT) and telomerase RNA (TR). In the complex, TR serves as the template for TERT, which catalyzes the addition of telomeric repeats (TTAGGG)_n_ at chromosome ends [3]. Vertebrate TRs exhibit a universally conserved secondary structure comprised of four structural domains (Fig. 1): the pseudoknot (core) domain containing the template sequence in conserved region (CR) 1 (CR1), the CR4 and CR5 domains with a highly conserved stem-loop structure (CR4-5), the H/ACA box domain, and the CR7 domain [4]. CR1 encodes the template sequence that is utilized for the extension of the telomeric repeats, while the CR4-5 domain contributes to the processivity of telomerase and is essential for stable assembly with TERT. The H/ACA box and CR7 domains confer TR stability [4]–[6].

Telomerase activity is absent in most somatic cells, but commonly up-regulated in rapidly dividing cells including transformed cells [7]. Consistent with this observation, telomerase activity is significantly elevated in over 85% of human cancers and over 70% of immortalized human cell lines [8]. The absence of telomerase activity often leads to progressive telomere shortening resulting in cellular senescence and irreversible cell cycle arrest [9]. Several tumor-inducing viruses have evolved strategies to evade or subvert mechanisms controlling cellular senescence, mainly via the up-regulation of TERT, which is generally the limiting factor for telomerase activity [10]–[13]. It has been suggested that up-regulation of TERT expression and, consequently, increased telomerase activity ensures the proliferative potential of persistently infected cells.

One of the most efficient viruses with respect to induction of fatal tumors is Marek's disease virus (MDV). MDV is a lymphotropic herpesvirus that causes a well-described syndrome, Marek's disease (MD), in chickens. MD is characterized by neurological disorders, immune suppression, and malignant T cell lymphomas [14]. The rapid onset of lymphomas developing within 2 to 3 weeks post-infection (p.i.) and high tumor-induced mortalities of 90–100% in susceptible chickens make MDV-induced transformation an ideal model to study virus-induced tumorigenesis in a natural virus-host setting [15]. A number of MDV-encoded genes have been shown to be involved in MDV-induced transformation. The major MDV oncogene, *meq*, encodes a basic leucine zipper (bZIP) transcription factor similar to the cellular homologues c-Jun, c-Fos, and c-Myc. Meq dimerizes with other bZIP transcription factors and modulates expression of both cellular and viral genes [16], [17]. MDV also encodes other genes products and sequence elements, which perform auxiliary functions in transformation [18]. One such element is a TR homologue termed viral TR (vTR) that shares 88% sequence identity with chicken TR (chTR) [19]. The high sequence homology suggests vTR was likely acquired from the chicken genome during virus-host co-evolution. Compared to its cellular counterpart, chTR, interaction of vTR with TERT results in higher telomerase processivity [20], [21]. It was shown that vTR contributes to the rapid onset of lymphoma formation by serving as a template for TERT, but it also has functions that are independent of the telomerase complex. It is predominantly the telomerase-independent functions of vTR that are responsible for tumor progression and dissemination [21], [22].

*In vitro* experiments demonstrated that mutations in the template sequence within CR1 of human and mouse TR can result in telomere instabilities, aberrant chromosome separation and segregation, and ultimately apoptosis [23], [24]. TRs with a mutated template can induce unique checkpoint responses that are different from DNA damage or loss-of-telomerase responses, even at low mutant TR expression levels and in the presence of wild-type TR. In addition, pro-apoptotic effects were also shown for TRs harboring mutant template or oligonucleotides specifying mutant template sequences and such molecules are discussed as anti-tumor therapeutics in different types of cancers [23], [25], [26].

Here, we investigated the effect of a mutant vTR template sequence (AU5) on the tumor-promoting capacity of a highly oncogenic avian herpesvirus in its natural host. Mutation of the template sequence of MDV-encoded vTR completely abrogated virus-induced tumor formation in chickens. Introduction of a second mutation in the stem loop (CR4-5) region that abolishes a functional interaction of vTR with TERT restored lymphomagenesis, confirming that the abrogation of tumorigenesis shown for the mutant virus is dependent on telomerase activity through interaction of mutant vTR and TERT. In vaccination-challenge studies, the virus expressing mutant template protected chickens from lethal challenge with a very virulent MDV strain.

## Results

### Expression of mutant template sequence vTR significantly reduces proliferation of avian cancer cells *in vitro*

**Figure 2:**
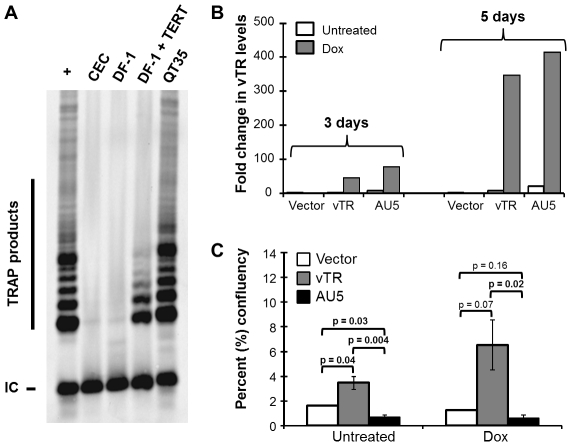
Expression of vTR harboring the mutant template (AU5) decreases cell proliferation of an avian cancer cell line. A) Analysis of telomerase activity in primary CEC cultures, the chicken fibroblast cell line DF-1 with or without TERT expression, and the quail QT35 cancer cell line using TRAP assays. TRAP products representing telomere elongation and internal control (IC) are indicated. B) RT-qPCR of vTR copies in polyclonal empty vector, vTR or AU5 QT35 cell lines induced with 1 µg/ml doxycycline (Dox) for 3 and 5 d or left uninduced. Data is shown as relative quantitation (RQ) of vTR copies relative to quail GAPDH RNA copies that served as an endogenous control. C) Percent (%) confluency of vector, vTR, and AU5 cell lines over the course of 31 d. Results are shown as means and standard errors of three independent experiments. *P* values were determined between each group using Student's *t* tests.

TRs harboring mutations in the template sequence were previously shown to result in telomere instabilities, aberrant chromosome separation and segregation, and ultimately apoptosis in mammalian cells *in vitro* [23]. Led by these previous observations, we hypothesized that expression of vTR encoding a mutated template sequence (AATCCCAATC to A**TATAT**A**TAT** ), termed AU5, could have an effect on avian cancer cells that is similar to that described previously for mammalian cells [23], [24]. In order to test our hypothesis, we first screened a number of avian primary cells and permanent cancer cell lines to determine the optimal system that would provide sufficient levels of telomerase activity. We performed telomere repeat amplification protocol (TRAP) assays to detect telomerase activity in primary chicken embryo cell (CEC) cultures, the chicken fibroblast cell line DF-1 [27], and the quail cancer cell line QT35 [28]. CEC and DF-1 cells did not exhibit telomerase activity, while the QT35 cancer cell had high telomerase activity as evidenced by the presence of numerous TRAP products (Fig. 2A). A DF-1 cell line stably expressing TERT showed some telomerase activity, suggesting that TERT was the limiting factor for telomerase activity in this cell line. Based on the results, we used a previously established QT35 cancer cell line that allowed tetracycline-inducible expression [29] of vTR or vTR-AU5 (AU5). During the establishment of cell lines we observed that even un-induced AU5 cell lines replicated markedly slower. From many initial clones, only a single monoclonal AU5 cell line could be established, suggesting a strong selection against leaky AU5 expression. This effect has been previously observed during the development of mammalian cell lines expressing TR template mutants [23]. Therefore, polyclonal vTR and AU5 cell lines were used to determine the effect of AU5 expression on cancer cell proliferation. RT-qPCR analysis of polyclonal cells, confirmed leaky expression of the constructs and that vTR and AU5 expression could be increased by more than 300-fold upon induction with doxycycline after 5 days of treatment (Fig. 2B).

To determine if AU5 inhibits cancer cell proliferation, we analyzed colony formation by measuring confluency over 31 days in the presence or absence of doxycycline. Constitutive or induced expression of wild-type vTR resulted in enhanced proliferation when compared cells harboring the vector control as described previously [21]. In contrast, cell lines harboring AU5 exhibited a significant growth defect when compared to vTR and control cell lines (Fig. 2C). Increased expression of AU5 following induction resulted in only slightly reduced cell proliferation when compared with non-induced cells, suggesting that expression of only low levels of AU5 are sufficient to reduce growth of the QT35 cancer cell line. We concluded that our results are consistent with those of TR over-expression in human and murine cancer cells [23], [24] and show that expression of the MDV vTR can help stabilize and/or promote growth, while mutation of the template sequence significantly impairs proliferation of the avian QT35 cancer cell line.

### Mutation of the vTR template sequence abrogates MDV-induced lymphomagenesis

**Figure 3:**
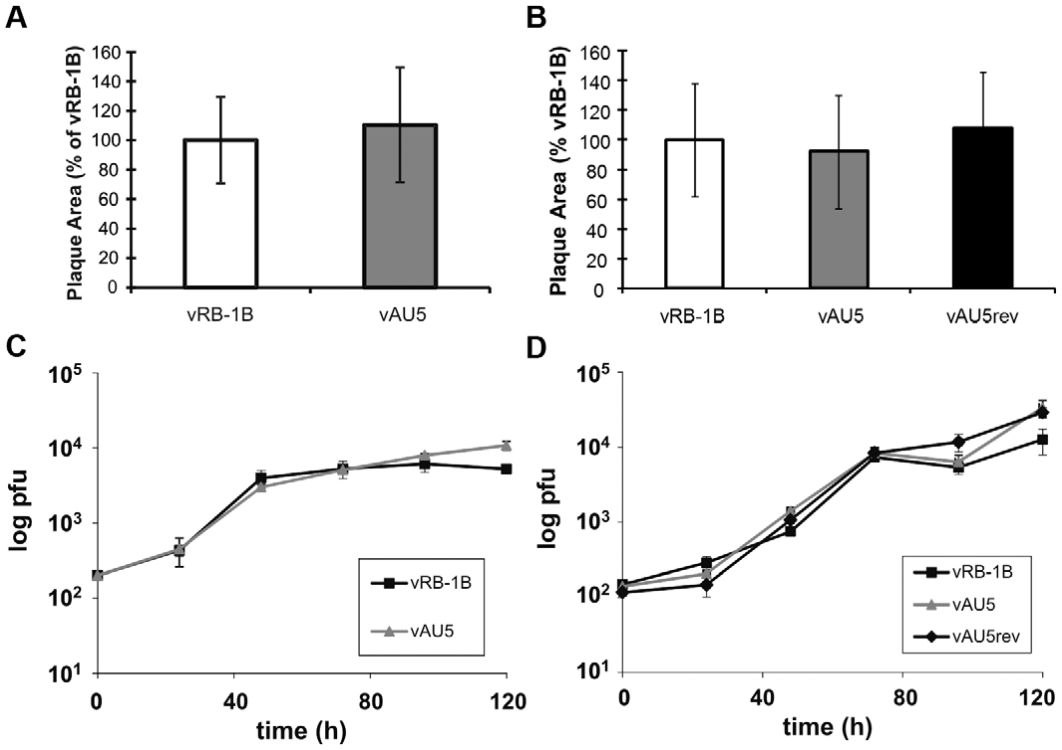
MDV harboring a template mutant vTR replicate comparable to parental and revertant viruses *in vitro*. A–B) Plaque areas were determined for 35 (A) or 100 (B) randomly selected plaques for indicated viruses. Results are shown as mean plaque areas in percent of the parental vRB-1B with standard deviations (error bars). C–D) Multi-step growth kinetics of indicated viruses were performed in triplicates and are shown as means with standard deviations (error bars).

Since mutation of the template sequence of vTR resulted in decreased proliferation of QT35 cancer cells (Fig. 1C), we hypothesized that the mutation in the context of virus infection may have an effect on MDV replication and tumorigenesis *in vivo*. Therefore, we mutated the template sequence of vTR (AU5) in pRB-1B, an infectious bacterial artificial chromosome (BAC) clone of the highly oncogenic RB-1B MDV strain using two-step Red-mediated recombination [30], [31]. Two rounds of mutagenesis allowed the desired alteration of both copies of the diploid vTR gene within the MDV genome, and transfection of the recombinant BAC clone into CEC resulted in the reconstitution of the vTR template mutant virus (vAU5). Furthermore, a revertant clone (AU5rev) was generated in which the wild-type template sequence was restored in the mutant vAU5. Following virus reconstitution, we performed plaque size assays and multi-step growth kinetics in CEC that revealed that the growth properties of vAU5 were indistinguishable from those of parental (vRB-1B) and revertant (vAU5rev) viruses (Fig. 3A–D).

**Figure 4:**
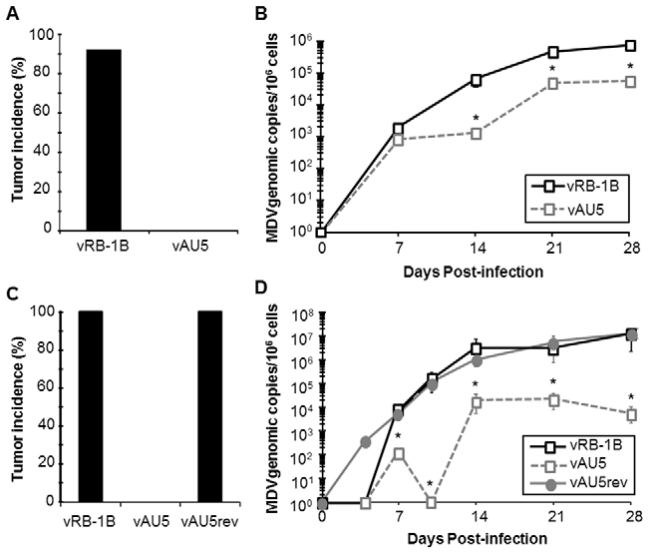
Tumor induction and *in vivo* replication of MDV harboring mutant template sequence (AU5) vTR. MD-susceptible chickens were inoculated with 1,000 PFU of either vRB-1B (n = 12) or vAU5 (n = 11) in experiment 1 (A and B) and 2,000 PFU of vRB-1B (n = 17), vAU5 (n = 19), or vAU5rev (n = 17) in experiment 2 (C and D). A and C) Necropsies were performed on chickens following onset of clinical signs of MD during both experiments and the percent of infected chickens developing tumors over 13 wk was determined. B and D) DNA was obtained from peripheral blood of chickens infected with each respective virus and viral genome copies were determined using qPCR assays. MDV ICP4 copies were normalized to the chicken iNOS gene and are shown as MDV genome copies per 1×10^6^ cells with standard error of mean bars. Viremia induced by vAU5 was significantly reduced when compared to vRB-1B (in experiment B) 14dpi, p = 0.007; 21dpi, p =  0.003; 28dpi, p = 0.014; in experiment D) 14dpi, p = 0.013; 21dpi, p = 0.004; 28dpi, p = 0.049) and vAU5rev (in experiment D) 10dpi, p = 0.022; 14dpi, p = 0,003) at the time points indicated by asterisks (*) using Student's *t* tests.

Next, we determined if expression of AU5 had an effect on MDV replication, disease, and tumor incidence *in vivo*. In two independent experiments, we infected 1-day-old P2a chickens with vRB-1B, vAU5, or vAU5rev and monitored virus levels in the blood using qPCR assays until 28 days post infection (dpi). vAU5 replication was significantly impaired when compared to parental and revertant viruses, indicating that the number of infected B and T cells is reduced (Fig. 4B and D). Consistent with the reduction of viremia, none of the chickens infected with vAU5 developed tumors in two independent experiments (0/10; 0/18) over the course of 13 weeks while parental (vRB-1B) or revertant (vAU5rev) viruses induced lymphomas in 92–100% of infected animals (Fig. 4A and C). We concluded from our data that expression of vTR harboring the AU5 mutation by MDV can completely abrogate virus-induced tumorigenesis in highly susceptible chickens, most likely by the elimination of MDV-infected and/or transformed cells by apoptosis.

### Abrogation of MDV-induced lymphomagenesis caused by expression of mutant vTR is dependent on its interaction with TERT

**Figure 5:**
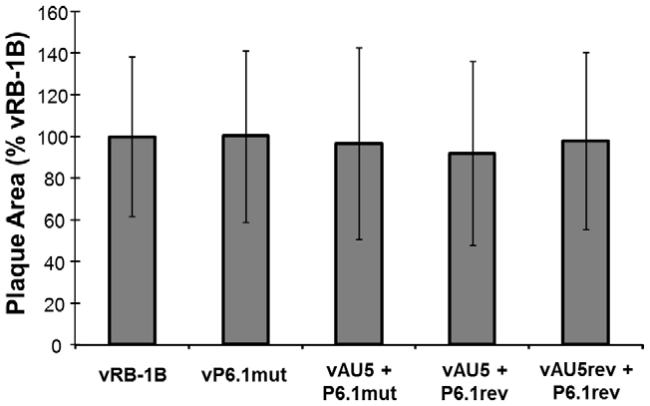
*In vitro* replication of parental, mutant, and revertant viruses. Plaque areas were determined for 100 randomly selected plaques for the indicated viruses. Results are shown as mean plaque areas in percent of the parental vRB-1B with standard deviations (error bars).

**Figure 6:**
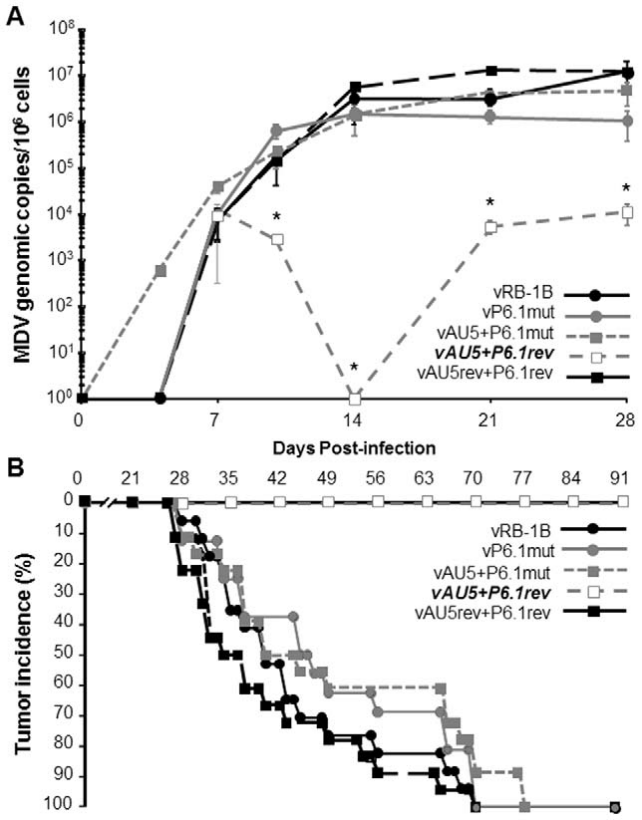
Secondary mutation of the vTR-TERT interaction domain, P6.1, rescues MDV replication and lymphomagenesis. MD-susceptible chickens were infected with vRB-1B (n = 17), vP6.1mut (n = 16), vAU5 + P6.1mut (n = 18), vAU5 + P6.1rev (n = 19), or vAU5rev + P6.1rev (n = 18). A) DNA was obtained from blood of infected chickens and MDV genome copies are shown per 1×10^6^ cells as in Fig. 3. Significant differences in genome copies between vAU5 + P6.1rev and vRB-1B (14dpi, p = 0.013; 21dpi, p = 0.004; 28dpi, p = 0.049) and vAU5rev + P6.1rev (14dpi, p = 0.002) are indicated with an asterisk (*) using Student's *t* test. B) Tumor incidences for each group infected with viruses contained only the AU5 mutation (empty boxes), additional/exclusively the P6.1 mutation (grey symbols) or parental and complete revertants (black symbols) were measured for 13 weeks.

We previously demonstrated that a mutation within the vTR P6.1 stem-loop can prevent incorporation of vTR into the telomerase complex and abolish enzymatic activity and telomere elongation [22]. To confirm that the absence of lymphoma in vAU5-infected animals was dependent on the presence of AU5 in the telomerase complex, we constructed mutant viruses in which the AU5 and P6.1 mutations were introduced into vTR either individually or together. Revertant viruses of each mutation were also generated. All constructed viruses replicated with kinetics comparable to those of parental and revertant viruses *in vitro* (Fig. 5). Upon infection of chickens with the recombinant viruses, qPCR analysis revealed that insertion of the P6.1 mutation into vAU5 (vAU5+P6.1mut) restored lytic virus replication to levels comparable to those of parental vRB-1B, while mutant virus only harboring the AU5 mutation (vAU5+P6.1rev) was significantly impaired in replication (Fig. 6A).

Like vAU5, vAU5+P6.1rev did not induce tumors in any of the infected chickens (0/19) (Fig. 6B). Two of the 19 chickens (11%) died over the course of the 13 week experiment, which was likely due to immunosuppression and generalized wasting, common characteristics of MD and observed in earlier reports using viruses that are unable to express vTR [21]. Viruses that contained the P6.1 and the AU5 mutation (vAU5+P6.1mut) induced lymphomas in 100% of infected animals. Furthermore, vP6.1, parental and complete revertant viruses caused tumors in all animals infected with the respective viruses. From the results we concluded that abrogation of lymphoma formation after infection with an MDV specifying the AU5 template mutation is indeed dependent on the interaction of template mutant vTR with TERT and has no effect if it is not incorporated into the telomerase complex.

### Vaccination with vTR template mutant virus confers protection against lethal MDV challenge

**Figure 7:**
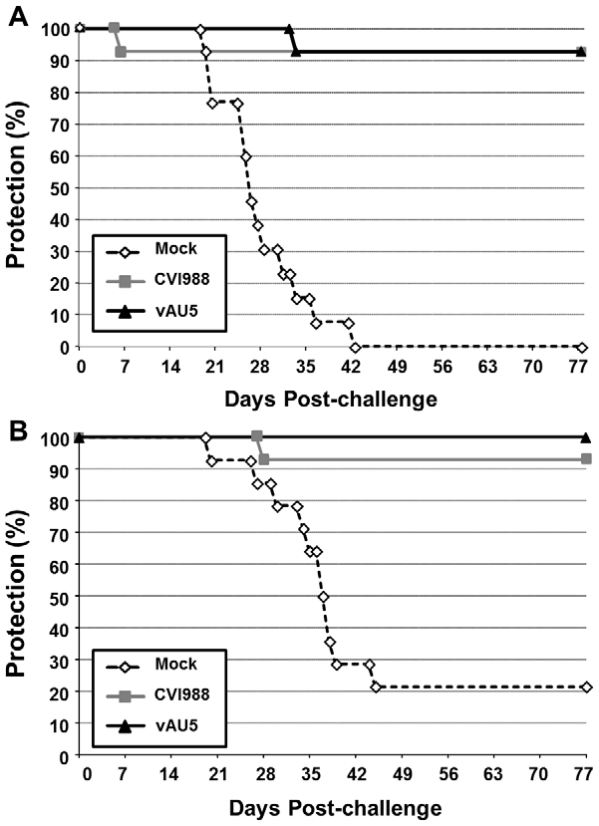
Immunization with the vAU5 mutant viruses protects chickens from lethal MDV infection. A) MD-incidence in N2a (A; n = 14) and B) P2a (B; n = 14) chickens vaccinated with either vAU5, CVI988 or media alone (Mock) before challenge-infection with RB-1B. Precent protection from the onset of disease or being tumor-positive at termination of the experiment is shown in % of the animals.

Since MDV harboring the template mutant vTR did not induce tumors but still replicated in chickens, we addressed the question whether vAU5 could induce a robust enough immune response to serve as a vaccine. Groups of 1-day-old P2a (highly susceptible to MD) and N2a (partially resistant to MD) chickens were inoculated with diluent, vAU5, or the widely used, commercial vaccine strain CVI988. Vaccinated chickens were challenged 10 days later with the very virulent RB-1B MDV strain. Chickens receiving the diluent developed tumors with expected frequencies of 100% in the P2a chickens and 79% in the N2a chickens after 13 weeks (Fig. 7A and B) [32]. vAU5 vaccinated N2a chickens were completely protected from lethal challenge, while 7% of the animals vaccinated with the commercial CVI988 vaccine strain developed disease with a protective index of 91%. In P2a chickens that are highly susceptible to MD, both vAU5 and CVI988 efficiently induced protection against challenge infection, as only 1 animal in each group developed disease. The protective index of vAU5 and CVI988 in P2a animals was 93% and 92%, respectively. These results suggest that mutation of the template region of vTR in a virulent MDV can serve as a strategy to induce protection against virus-induced lymphomas.

## Discussion

We here report on effects of a mutation in the template sequence (CR1) of vTR encoded by MDV on virus replication and tumorigenesis in a natural virus-host model. Mutation of the vTR template sequence from AATCCCAATC to A**TATAT**A**TAT** (AU5) resulted in decreased proliferation of the QT35 avian cancer cell line (Fig. 2), as had been described for TR in mammalian cells [23], [24]. Introduction of the template sequence mutation in vTR in the context of the viral genome and infection of MD-susceptible chickens with mutant virus (vAU5) resulted in complete absence of tumors and low-level viral replication *in vivo* (Fig. 4). Secondary mutation of the vTR stem-loop sequence (P6.1), abolishing the interaction of mutant vTR with TERT, restored virus-induced tumorigenesis (Fig. 6), thus showing that vTR-TERT interaction and functional telomerase activity is required for the anti-tumorigenic effects of the mutant template sequence in a viral background. Vaccination with MDV harboring the vTR template mutation not only abrogated herpesvirus-induced tumorigenesis, but also protected chickens from a lethal challenge with a very virulent MDV strain.

**Figure 8:**
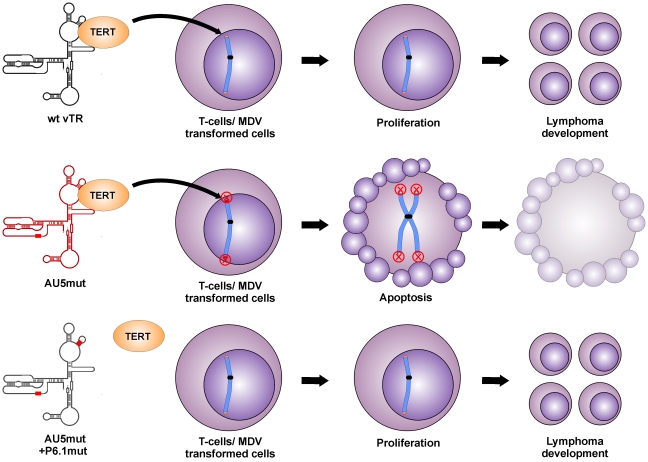
Proposed model for abrogation of tumor induction by mutant template sequence vTR through incorporation of mutant telomere sequences in transformed T cells. Expression of vTR AU5 leads to telomere instabilities, aberrant chromosome separation and segregation, and finally apoptosis induction in the presence of TERT (upper panel). Without vTR interaction with TERT by mutation of the P6.1 stem loop, mutant template sequences (AU5) are not incorporated in the telomeres of transformed cells and thus proliferation of transformed cells continues, leading to lymphomas.

We surmise that the reduced proliferation of QT35 expressing vTR with a template sequence mutation, as well as the absence of tumors and greatly reduced lytic replication in chickens are both caused by the incorporation of mutant telomeric repeat sequences into host telomeres of infected cells, which eventually leads to telomere crisis and apoptosis (Fig. 8). This sequence of events has been shown previously in other mammalian systems *in vitro*, where even low levels of mutant TR induced a unique checkpoint response resulting in telomere instabilities, aberrant chromosome separation and segregation, and apoptosis [23], [24]. In addition, the pro-apoptotic effect of TRs harboring mutant templates or oligonucleotides specifying mutant template sequences has also been shown [23], [25], [26].

It is interesting to note that the QT35 cancer cell line was previously shown to maintain MDV in a latent state [33] and that the cells express MDV vTR at very low levels (Fig. 2B). Despite the expression of endogenous quail TR and MDV vTR, AU5 expression had a negative effect on the replication of QT35 cancer cells. Consequently, induced over-expression of wild-type vTR led to increased proliferation of QT35 cancer cells, further lending support to the interpretation that vTR performs an important function in the early maintenance of transformed cells [21]. Induced expression of the AU5 sequence leading to incorporation of mutant template sequences significantly reduced proliferation presumably by inducing apoptosis, again consistent with previous studies on mammalian TRs [23].

vTR was previously shown to contribute to MDV-induced lymphomagenesis. Deletion of vTR in the MDV genome resulted in significantly reduced tumor incidences but the mutation did not affect virus replication *in vivo* [21]. Mutation of the vTR template region (AU5) in MDV, however, completely abrogated tumorigenesis and reduced viral loads in infected animals, likely via inhibition of cancer cell replication through induction of apoptosis. vTR has at least two functions during lymphomagenesis, one that is dependent and one that is independent of telomerase activity. The telomerase-dependent function plays an important role in the early onset of disease but is dispensable for tumorigenesis. This conclusion is supported by studies showing that MDV harboring a mutated vTR incapable of interaction with TERT (P6.1mut) can still induce tumors in chickens, albeit resulting in a delayed onset of tumor formation [22]. vTR functions that are independent of its presence in the telomerase complex seem to be important for lymphomagenesis but are poorly understood. We utilized this previously published mutation to determine if the induction of apoptosis and abrogation of tumorigenesis is dependent on the incorporation of AU5 into the telomerase complex. While MDV harboring AU5 vTR are incapable of inducing tumors, mutation in the vTR-TERT interaction domain (vAU5+P6.1mut) in vAU5 completely restored tumorigenesis.

We therefore concluded that restored ability of vAU5+P6.1mut to induce tumors is presumably caused by the inability of the telomerase to incorporate mutant telomeric repeats at the ends of host chromosomes and cause telomere crisis and apoptosis; hence the pro-oncogenic functions of vTR that are independent of the telomerase complex prevail. Our results demonstrate that vAU5+P6.1mut can efficiently cause lymphoma, which confirms that vTR has tumor-promoting functions independent of the telomerase complex and that they are mediated by a vTR domain outside of the template region.

The fact that vAU5 was unable to induce tumors in highly susceptible P2a chickens suggested that it could serve as a potential vaccine against MD. Vaccination with vAU5 protected chickens from lethal challenge infection with the very virulent MDV strain RB-1B at least as efficiently as the commercial vaccine strain CVI988/Rispens that is commonly used in the field [34]. However, the residual mortality observed in some chickens has to be clarified to ensure the safety of the vaccine candidate. A similar phenomenon of low levels of mortality in highly susceptible birds, similar to the one observed here, was also observed with MDV mutants in which the major oncoprotein of MDV, Meq, was absent. Infection with *meq* deletion viruses did not cause tumors [35], but severe lymphoid atrophy and immunosuppression was evident [36]. Likely, a combination of vTR template mutation with other modifications in the MDV genome targeting genes important for replication could therefore increase the safety of the vaccine and prevent lymphoid atrophy. Here, we suggest a new strategy that could be applied to the next generation of MD vaccines, which will certainly be needed because recently isolated MDV strains are capable of evading immune protection provided by current vaccines [15], [37], [38].

## Materials and Methods

### Ethics statement

This study was carried out in strict accordance with the recommendations in the Guide for the Care and Use of Laboratory Animals of the National Institutes of Health. The protocol was approved by the Committee on the Ethics of Animal Experiments of Cornell University (permit number 2002-0085 and 2008-0018). The animal care facilities and programs of Cornell University meet the requirements of the law (89–544, 91–579, 94–276) and NIH regulations on laboratory animals, and are in compliance with the Animal Welfare Act, PL 279. All experimental procedures were in compliance with approval of Cornell University's Institutional Animal Care and Use Committee (IACUC) and all efforts were made to minimize suffering.

### Generation of mutant MDV

Recombinant viruses were generated by two-step Red-mediated recombination as previously described [30], [31]. Primers used for construction of template sequence (CR1) AU5 mutants (vAU5) and revertants (vAU5rev) are shown in Table 1. Primers used for construction of the P6.1 vTR-TERT interaction domain mutants and revertants have previously been published [22].

**Table 1:** Primers used for cloning and generation of mutant and revertant AU5 constructs.

Construct name		sequence (5′ → 3′)
**AU5**	for	GTTCCCCCGGCACACGTGGCGGGTGGAAGGCTCCGCTGTGT**TATATATATA**ACGGAGGTATTGATGTAGGGATAACAGGGTAATCGATTT
	rev	GGCGGAGGGAGCGCGGCGACAGTACCATCAATACCTCCGT**TATATATATA**ACACAGCGGAGCCTTCCGCCAGTGTTACAACCAATTAACC
**AU5rev**	for	GTTCCCCCGGCACACGTGGCGGGTGGAAGGCTCCGCTGTGT**CTAACCCTAAT**CGGAGGTATTGATGGTAGGGATAACAGGGTAATCGATTT
	rev	CGGGCGGAGGGAGCGCGGCGACAGTACCATCAATACCTCCG**ATTAGGGTTAG**ACACAGCGGAGCCGCCAGTGTTACAACCAATTAACC

Underlined sequence indicates the template binding region of the primers for PCR amplification with pEPKanS. Bold indicates the sequences mutated or reverted to parental sequences.

### Propagation of MDV

CEC cultures were prepared from 10-day-old specific-pathogen-free (SPF) embryos using standard methods [39]. Recombinant viruses were reconstituted from purified BAC DNA in CEC cultures using CaPO_4_ transfection [40]. The *lox*P flanked mini-F sequences within the infectious clones were removed by co-transfection with a *Cre* recombinase expression vector (pCAGGS-NLS/Cre) as previously described and screened via analytical PCR [30]. Virus propagation, plaque area measurements and multi-step growth kinetics were also performed as described previously [41].

### Animal studies

SPF P2a (MHC haplotype *B^19^B^19^*) or N2a (MHC haplotype *B^21^B^21^*) chickens were obtained from departmental flocks and housed in poultry isolation units. Chickens were inoculated with 1,000 or 2,000 plaque forming units (PFU) of virus by intra-abdominal injection and evaluated for symptoms of MD on a daily basis. Necropsies were performed on chickens showing clinical signs of MD, as well as all remaining chickens at the termination of the experiment.

### Chicken blood DNA extraction and qPCR assays

DNA was extracted from whole blood of eight chickens for each group randomly selected prior to the experiment and MDV genomic copies were determined by qPCR assays [41]. Briefly, MDV DNA copy numbers were detected using primers and probe specific for the MDV infected cell protein 4 (*ICP4)* locus that were normalized to cellular genome copies of chicken inducible nitric oxide synthase (*iNOS*).

### Cloning of vTR and AU5 Tet-on expression constructs and generation of stable cell lines

Tet-on constructs were generated by digestion of the pCMS-vTR and pCMS-vTR-AU5 constructs previously described [22] with EcoRI and XbaI. Resulting vTR or AU5 fragments were then cloned into the pcDNA4/TO/myc-his vector (Invitrogen, Carlsbad, CA) to generate pcDNA4/TO-vTR and pcDNA4/TO-AU5, respectively.

Inducible cell lines were generated based on QT35TR19, a previously described Tet-inducible QT35 cancer cell line (kindly provided by Karel A. Schat, Cornell University) and maintained as described previously [29]. To generate control, vTR, and AU5 expressing cell lines, QT35TR19 cells were transfected with pcDNA4/TO (empty vector), pcDNA4/TO-vTR, or pcDNA4/TO-AU5 using Lipofectamine2000 (Invitrogen, Carlsbad, CA) following the manufacturer's instructions. Monoclonal and polyclonal cell lines were selected with 5 µg/ml blasticidin and 500 µg/ml zeocin (Invitrogen). All cell lines were used between passage 5 and 10 in cell proliferation and RT-qPCR assays.

### Cell proliferation assays

Proliferation of Tet-inducible cell lines was evaluated as previously described [23]. Briefly, 2×10^3^ cells of each cell line were seeded into 35 mm dishes in triplicate and maintained in media with or without 1 µg/ml doxycycline with 2/3 media changed every 4–5 days. After 31 days, cells were fixed with 90% ice-cold acetone and stained with 1% crystal violet in 50% methanol. Percent confluency was determined using NIH ImageJ software by calculating the total area on the plates covered by cell colonies over the total area of the plate. The average % confluency was determined from three independent experiments.

### RT-qPCR assays for analysis of Tet-inducible vTR expression

One-thousand vTR, AU5, or empty vector Tet-inducible cells were treated with or without 1 µg/ml doxycycline in triplicate. After 3 and 5 days, total RNA was prepared using RNA STAT 60 as described previously [42]. Reverse transcription was performed using the ThermoScriptTM RT-PCR system (Invitrogen, Carlsbad, CA) with random hexamer oligonucleotides according to manufacturer's instructions.

Copies of vTR cDNA were determined by qPCR assays using the TaqMan Fast Universal Master Mix system (Applied Biosystems, Inc.) according to manufacturer's instructions and performed in an ABI Prism 7500 Fast Real-Time PCR System (Applied Biosystems, Inc.). Results were analyzed with the Sequence Detection Systems version V2.0.3 software using the comparative Ct method (2^−ΔΔCt^) of relative quantification. Primers and probe for the detection of MDV vTR and quail glyceraldehyde-3-phosphate dehydrogenase (GAPDH) that served as an endogenous control have been described previously [43], [44].

### Statistical analysis

Significant differences in % confluency assays and MDV replication using qPCR assays were determined using Student's *t* test or Tukey-Kramer comparison of means.
